# Supplementary material for: An AuNPs-Based Fluorescent Sensor with Truncated Aptamer for Detection of Sulfaquinoxaline in Water
Source: Biosensors (Basel). 2022 Jul 11;12(7):513. doi: 10.3390/bios12070513 (PMC9312917; doi:10.3390/bios12070513)
Supplement: Supplementary file 1 [file biosensors-12-00513-s001.zip › biosensors-1807016-supplementary.pdf]

Supplementary Material

An AuNPs-Based Fluorescent Sensor with Truncated Aptamer for Detection of Sulfaquinoxaline in Water

Xingyue Chen [1], Lulan Yang [1], Jiaming Tang [1], Xu Wen [1], Xiaoling Zheng [1], Lingling Chen [1], Jiaqi Li [1], Yong Xie [2,*] and Tao Le [1,*]

[1] College of Life Science, Chongqing Normal University, Chongqing 401331, China; chenxingyue31@163.com (X.C.); yang16086@126.com (L.Y.); tangjiamingt@163.com (J.T.); wenxu7968@163.com (X.W.); 2021110513064@stu.cqnu.edu.cn (X.Z.); cll2629593857@163.com (L.C.); lilijiaqi2020@163.com (J.L.)

[2] Bioassay 3D Reconstruction Laboratory, Chongqing College of Electronic Engineering, Chongqing 401331, China

* Correspondence: yongxie88@163.com (Y.X.); letao@cqnu.edu.cn (T.L.)

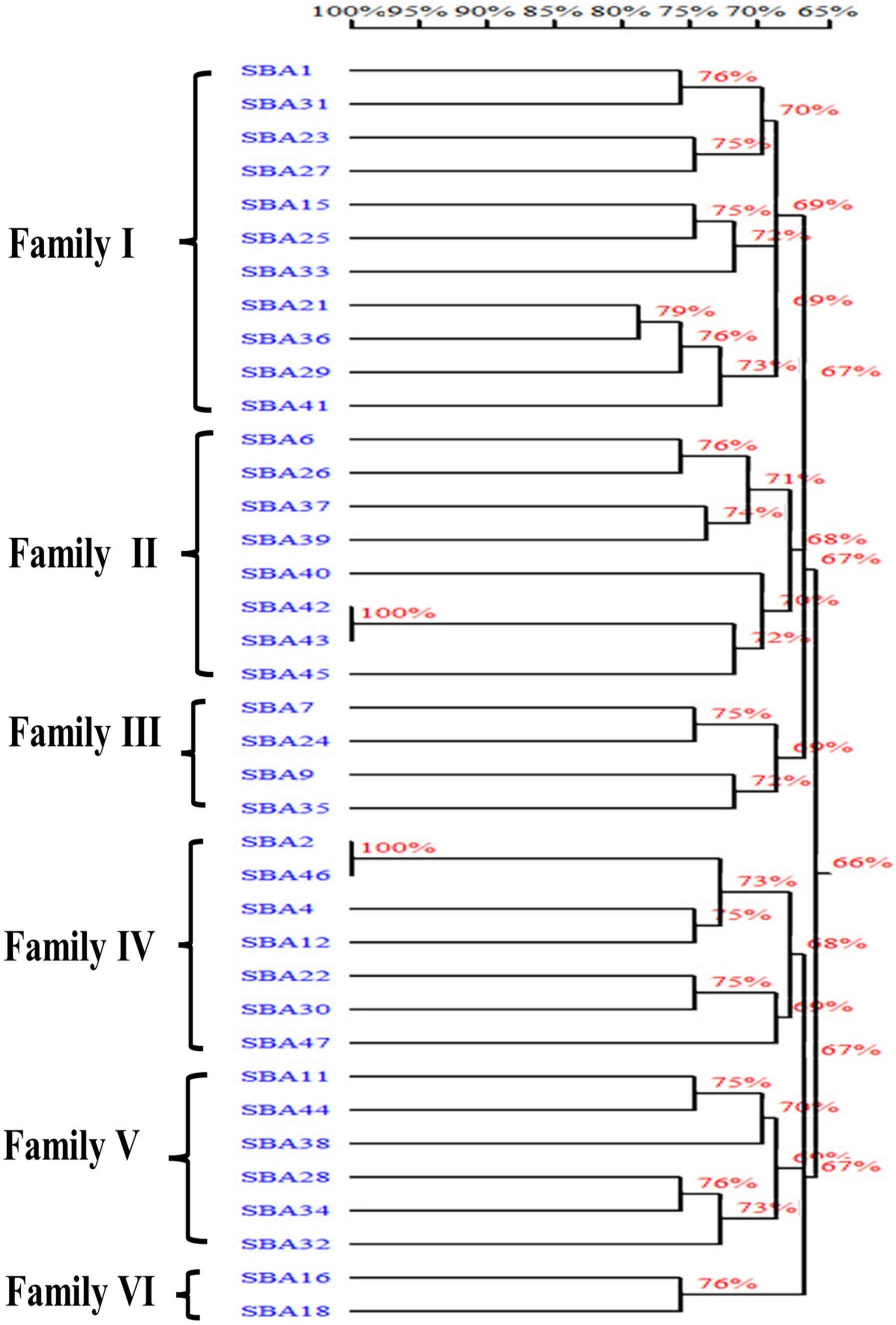

Figure S1 Phylogenetic tree of aptamers binding to SQX. These affinity sequences belong to 6 major families.

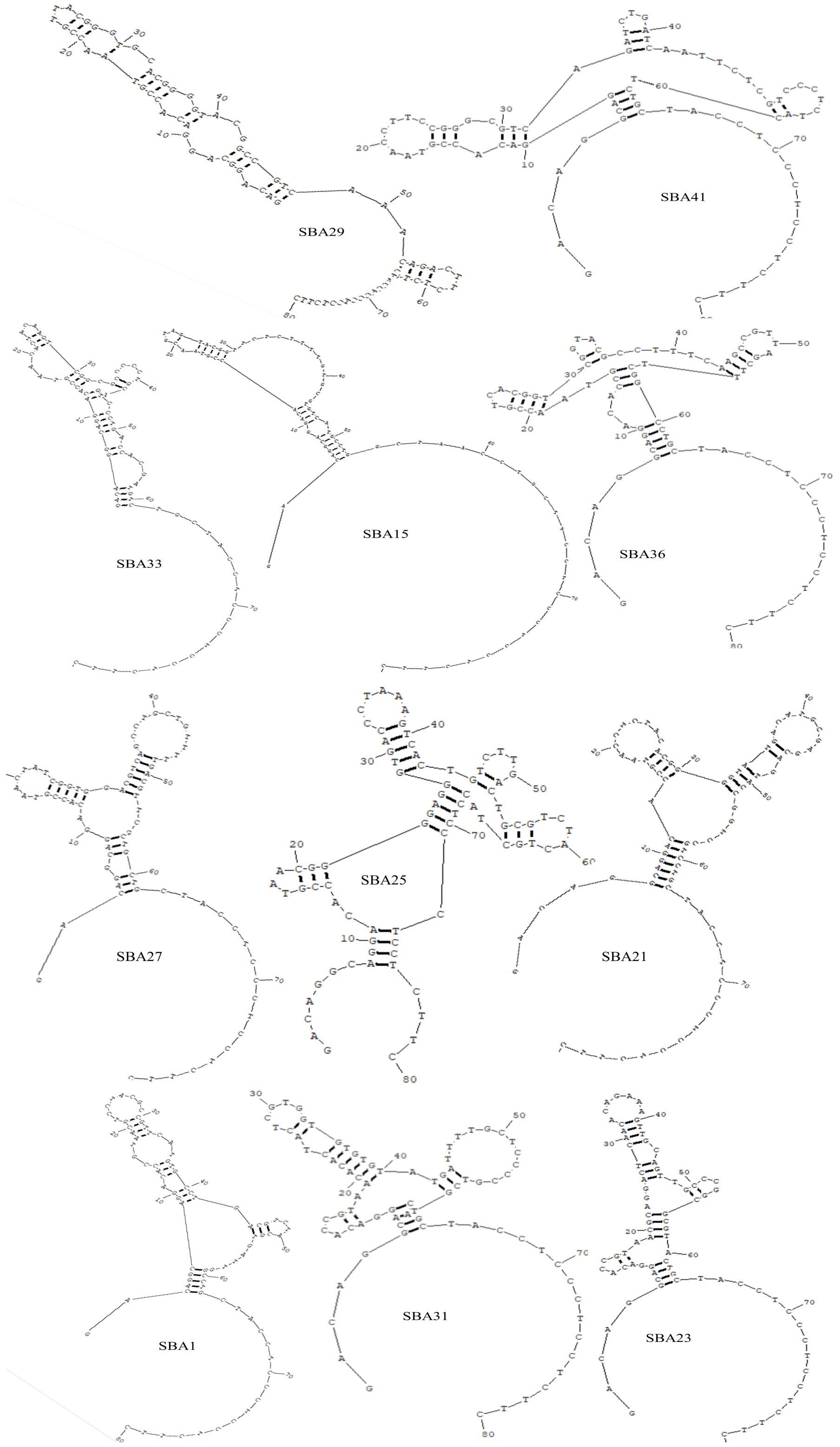

Figure S2 Secondary structure of aptamers in family I.

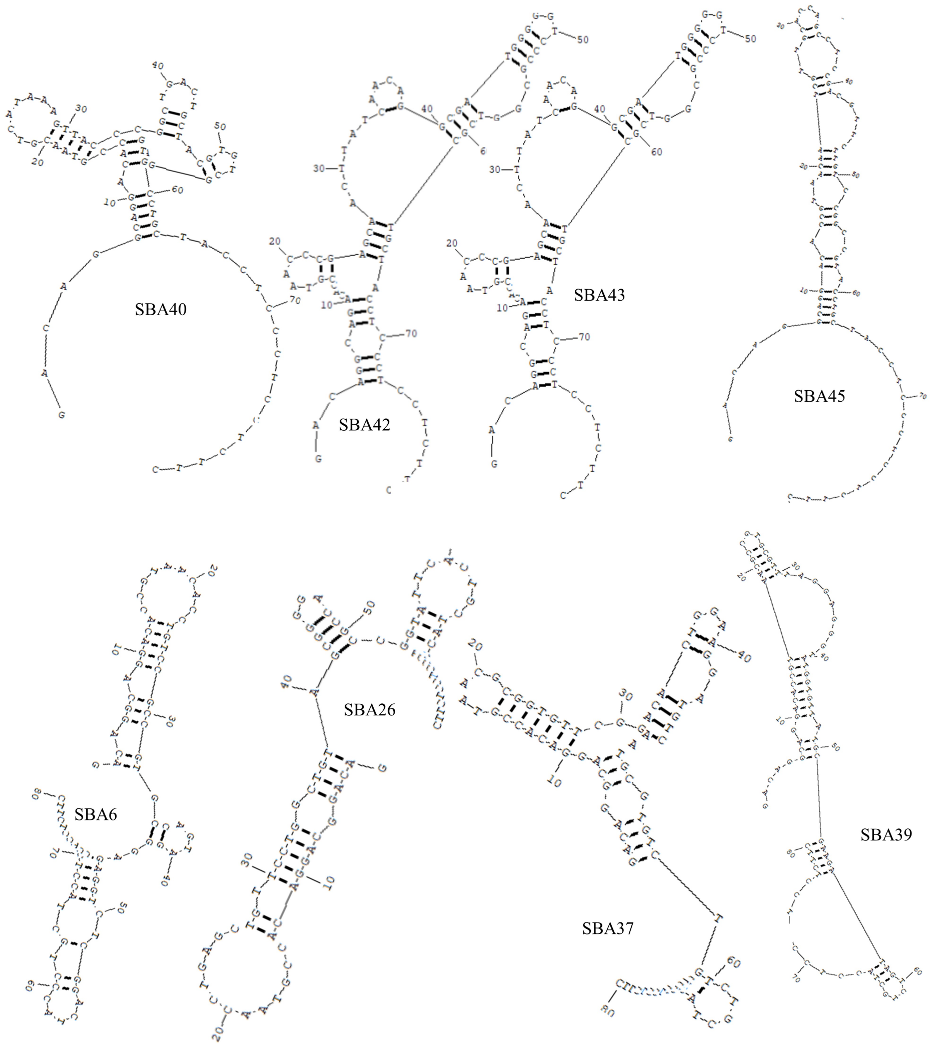

Figure S3 Secondary structure of aptamers in family II.

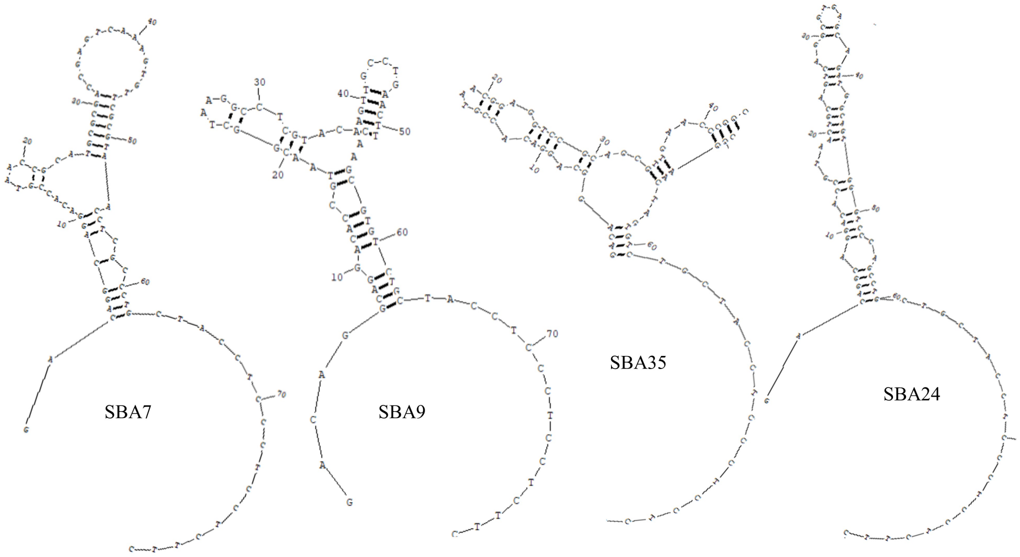

Figure S4 Secondary structure of aptamers in family III.

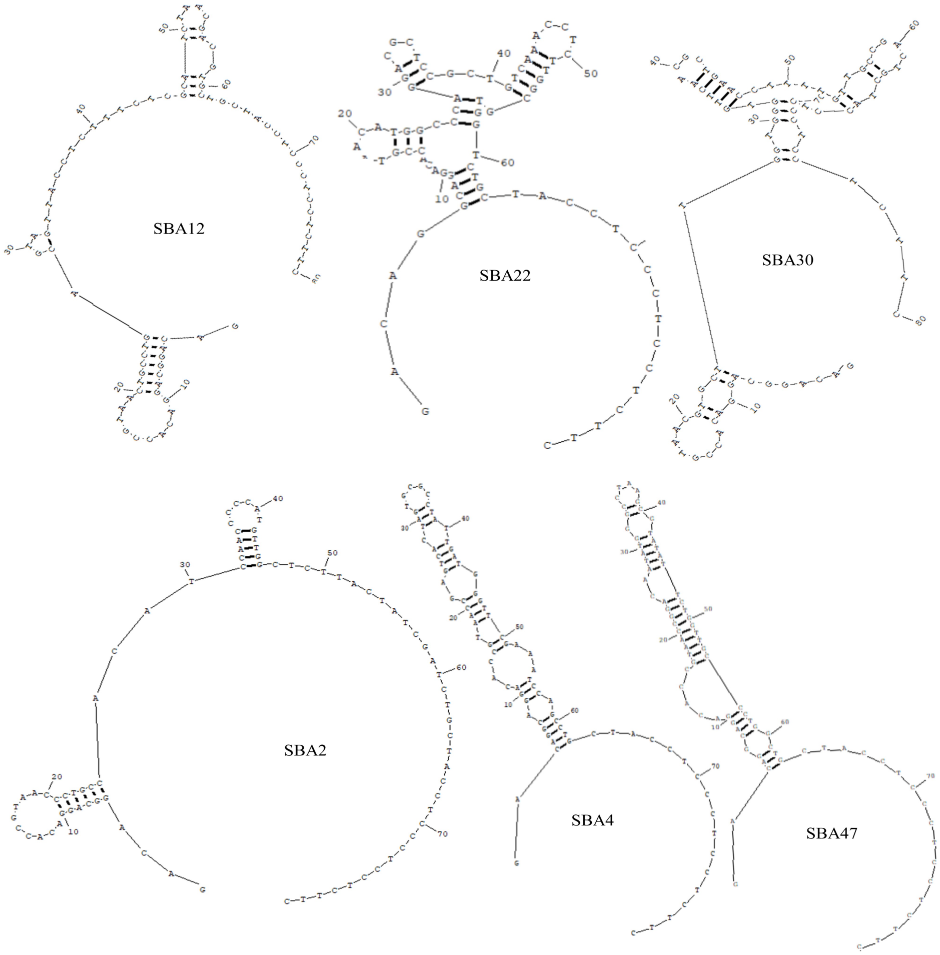

Figure S5 Secondary structure of aptamers in family IV.

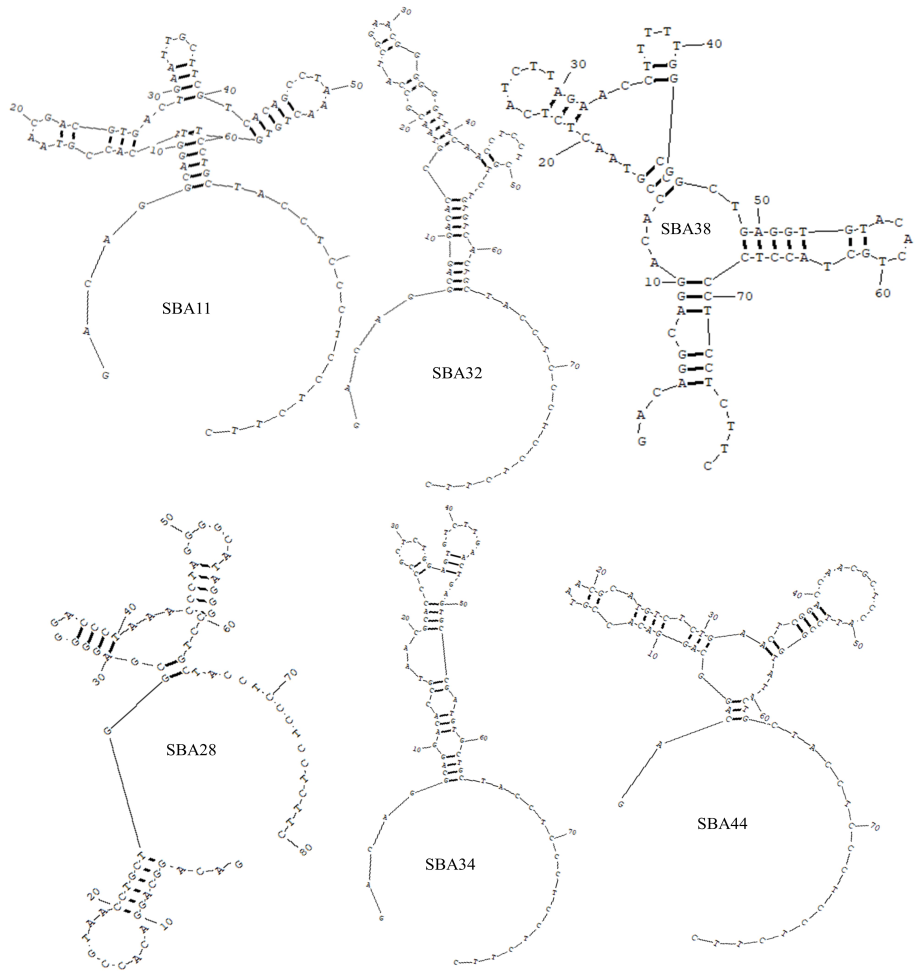

Figure S6 Secondary structure of aptamers in family V.

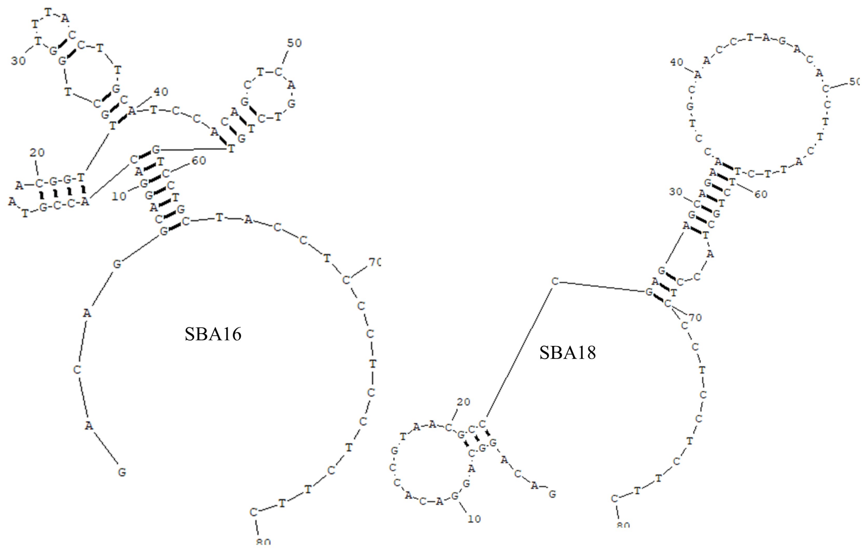

Figure S7 Secondary structure of aptamers in family VI.

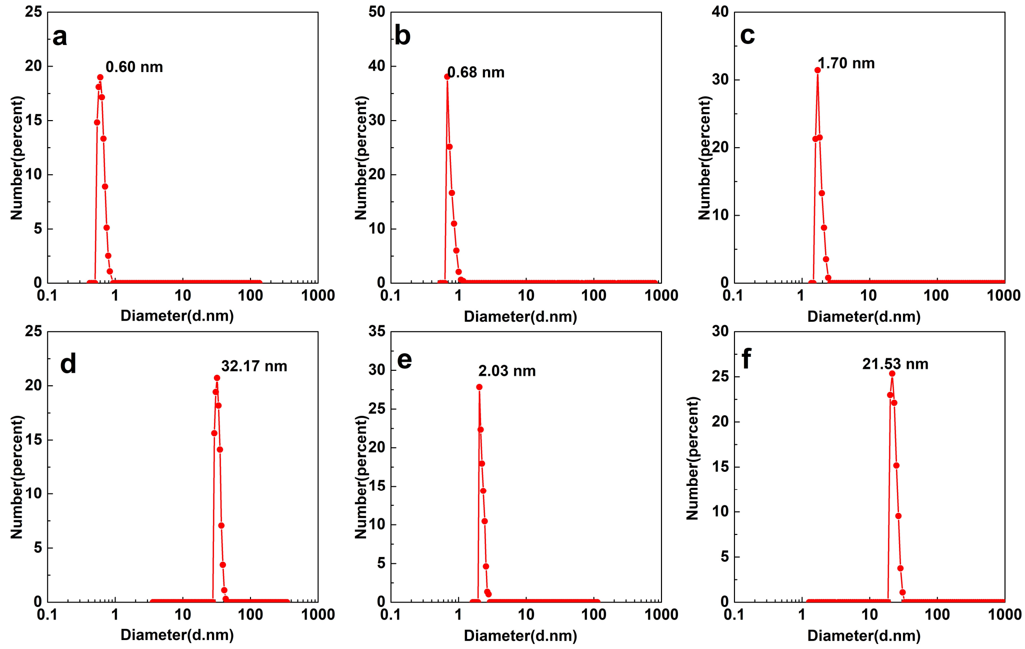

Figure S8 Dynamic light scattering (DLS) of AuNPs in different substances. (a) AuNPs; (b) SQX-AuNPs; (c) SBA28-1-AuNPs; (d) AuNPs-NaCl; (e) SBA28-1-AuNPs-NaCl; (f) SQX-SBA28-1-AuNPs-NaCl.

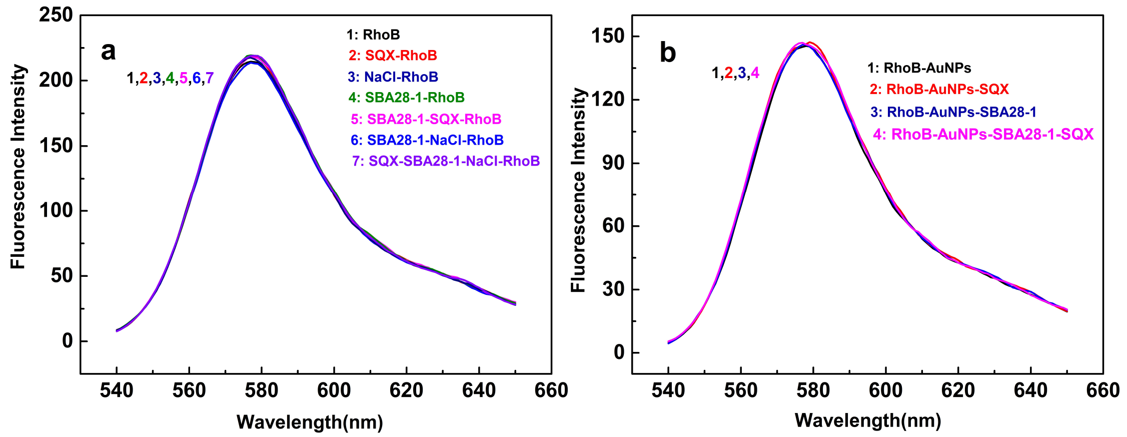

Figure S9 (a) Fluorescence spectra of RhoB with the absence of AuNPs in different sample solutions; (b) Fluorescence spectra of RhoB with the absence of NaCl in different sample solutions.

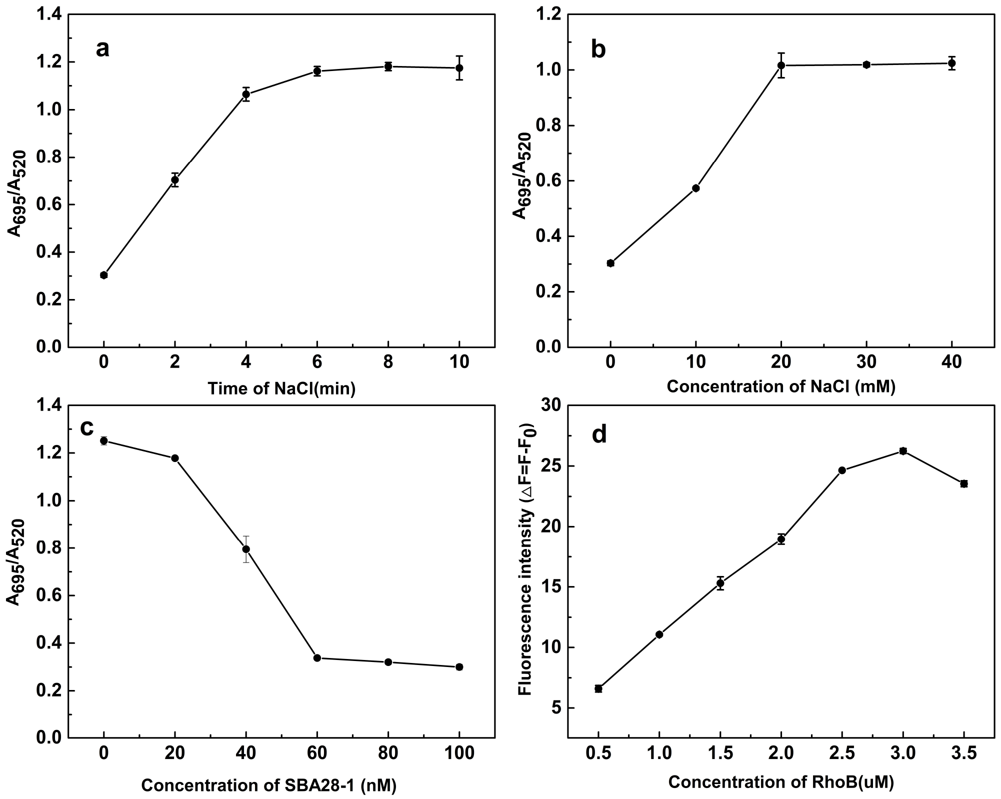

Figure S10 (a) The effect of NaCl incubation time on the absorbance ratio in the fluorescent aptasensor; (b) The effect of NaCl concentration on the absorbance ratio in the fluorescent aptasensor; (c) The effect of SBA28-1 concentration on the absorbance ratio in the fluorescent aptasensor; (d) The effect of RhoB concentration on the fluorescence intensity in the fluorescent aptasensor.
